# Propranolol Prevents Osteoporosis and up-regulates Leptin in Ovariectomized Rats 

**Published:** 2013

**Authors:** Xingguang Zhang, Xiaofeng Lv, Yanqi Zhang, Xiumin Jiao, Bin Chen

**Affiliations:** a *Beijing Military General Hospital, No. 5 Nan Men Cang, Dongcheng Distrct, Beijing 100700, P.R. China.*; b *China National Institute of Standardization*, *No. 4 Zhichun Road, Haidian District, Beijing 100088, P.R. China. *

**Keywords:** Propranolol, Osteoporosis, Leptin, Ovariectomized rat

## Abstract

Osteoporosis is a systemic skeletal disease and there is a close relationship between the sympathetic nervous system (SNS) and bone metabolism. Leptin has been shown to regulate bone formation and bone resorption via the SNS. However, the effect of SNS on leptin signaling has not been clearly understood. In the present study, we studied the effect of propranolol on ovariectomy-induced osteoporosis of rat. The results showed propranolol could increase the bone mass of ovariectomized rat. Propranolol could also up-regulate the level of peripheral leptin and the level of leptin receptor expression in ovariectomized rat hypothalamus. Our results indicate the effect of propranolol on ovariectomy-induced osteoporosis may be exerted, at least partly, through the regulation of leptin signaling and there may be an interaction between the SNS and leptin on the regulation of bone metabolism.

## Introduction

Osteoporosis is a systemic skeletal disease characterized by the loss of bone mass, microarchitectural deterioration of bone tissue, bone fragility and an increased risk of fracture. Usually, osteoporosis is considered to be an age-related health problem, especially in postmenopausal women. It has been reported that there is a close relationship between the sympathetic nervous system (SNS) and bone metabolism (1). Sympathetic activity increases significantly in postmenopausal women (2) and estrogen replacement therapy could reduce bone loss while sympathetic activity returns to normal. This has also been confirmed in ovariectomized rats (3). *β*2- adrenergic receptor has been found on osteoblasts and isoproterenol, a *β*-adrenergic agonist, leads to bone loss in mice. However, propranolol, the *β*-adrenergic antagonist, increases bone mass in mice (4), indicating the SNS could regulate the bone metabolism. In addition, the SNS can regulate some bone metabolism related factors, such as osteoclasts and bone marrow osteoclast marrow osteoclast differentiation factor (Rankl), osteoclast inhibitory factor (OPG) (5), calcitonin gene-related peptide (CGRP), P substance (SP) and tyrosine hydroxylase (TH) (6). 

Leptin is a protein hormone with important effects in regulating body weight, metabolism and reproductive function. It has been documented that leptin could regulate bone metabolism after binding to its receptor presumably on hypothalamic neurons. These effects are mainly through the activation of the SNS and subsequently decreasing bone formation and increasing osteoclast differentiation (4, 7, 8), while propranolol could block the bone loss caused by intracranial injection of leptin (4) and reserpine increases leptin mRNA levels in brown but not white adipose tissue in mice (9). However, the increase of peripheral levels can attenuate ovariectomy-induced osteoporosis in rats, suggesting the dual role of leptin and its receptors in regulating bone metabolism (10).

Although the above knowledge indicates that leptin modulates bone metabolism through the activation of SNS and more and more evidences suggest that low dose *β*-adrenergic antagonist can improve bone loss and bone fragility despite the difference of the osteoporosis models (11-14), the effect of SNS on leptin in osteoporosis has not been completely clarified. Therefore, we performed this study to investigate the role of β-blocker in the regulation of bone mineral density and leptin in ovariectomized rats.

## Experimental

*Animal studies*

Thirty 6-month-old female SD rats (250~290 g) (Experimental Animal Center of National Population and Family Planning Commission of P.P. China, Beijing, China) were randomly assigned into 3 groups: (1) Sham, (2) Ovariectomy (OVX) and (3) Propranolol treatment group (PRN). The rats in OVX and PRN groups were ovariectomized under anesthesia with 5% ketamine (100 mg·kg^-1^). Sham operations were performed by exposing the ovaries and excising a small adipose tissue. One week later, all the rats recovered very well without infection. Rats in OVX and PRN groups were administrated through gavage one week after the operation with saline or propranolol (8 mg·kg^-1^·d^-1^) daily for 11 weeks. All animals were allowed free access to food and water. Throughout the experiment, animals were maintained on a 12 h light/12 h dark cycle (lights on at 6:00 AM) at 22 °C with food and water available *ad libitum*. At the end of experiment, all the rats were sacrificed by cervical dislocation and approximately 2 mL of trunk venous blood was collected via eyeball enucleation. After the rats were killed, the bone mineral density (BMD) of left femoral and the 3^rd^, 4^th^ and 5^th^ vertebral was measured by dual energy X-ray absorptiometry (DXA; Excel Plus, Norland Corp.). The experiments were performed in accordance with the Helsinki Declaration of 1975. All the animals used in the study received humane care.

*RNA extraction and semi-quantitative RT-PCR*

Total RNA was extracted from hypothalamus with TRIzol reagent (Invitrogen, Carlsbad, CA, USA) according to the manual. The concentration and purity of total RNA were determined by a spectrophotometer (Eppendorf, Hamburg, Germany). Reverse transcription was performed using oligo (dT) primer RT mixtures with M-MLV (Promega, Madison, WI, USA) reverse transcriptase 200 U/20 μL, total RNA 1 μg / 20 μL. cDNA was stored at -20 °C. Primers used in the PCR reaction were as follows: β-actin, forward: 5’-CCT CTA TGC CAA CAC AGT GC-3’, reverse: 5’-GTA CTC CTG CTT GCT GAT CC-3’; rOb-R: forward: 5’ –CCA GCA CAA TCC AAT CA -3’, reverse: 5’-ACA TAG ACC GCA CAG AG -3’; rOb-Rb: forward: 5’-ACA CCT TAG ACC TCA CCA GTT T-3’, reverse: 5’-GAC TTC TTT TAG TGC CCC TTT T-3’. PCR reaction was performed with 2 μL of cDNA, 1 μg Taq DNA Polymerase (Fermentas, MBI, USA) and 0.5 μmol/L of forward and reverse primers, for a total volume of 20 μL. Reactions were started with a polymerase activation step at 94 °C for 3 min followed by 30 cycles of 94 °C for 20 sec, 55 °C for 30 sec 72 °C for 30 sec, and elongation at 72 °C for 5 min. The PCR products were electrophoresed on a 2% agarose gel in the presence of ethidium bromide, and absorbance were measured by densitometer. The ratio of targets to actin was used to evaluate the significance of the difference between various groups.

*Enzyme-linked immunosorbent assay*

The serum leptin levels were determined by Enzyme-linked immunosorbent assay (ELISA) with a commercial kit (Assay Design, Ann Arbor, MI, USA) according to the manufacturer’s instruction.

*Statistical analysis*

All the results are expressed as the means ± SD. Statistical significance was determined using SPSS 11.0 for Windows. One-way ANOVA was performed for multiple comparisons. Differences were deemed significant if the calculated P-value was < 0.05.

## Results

*General observations*

**Figure 1:**
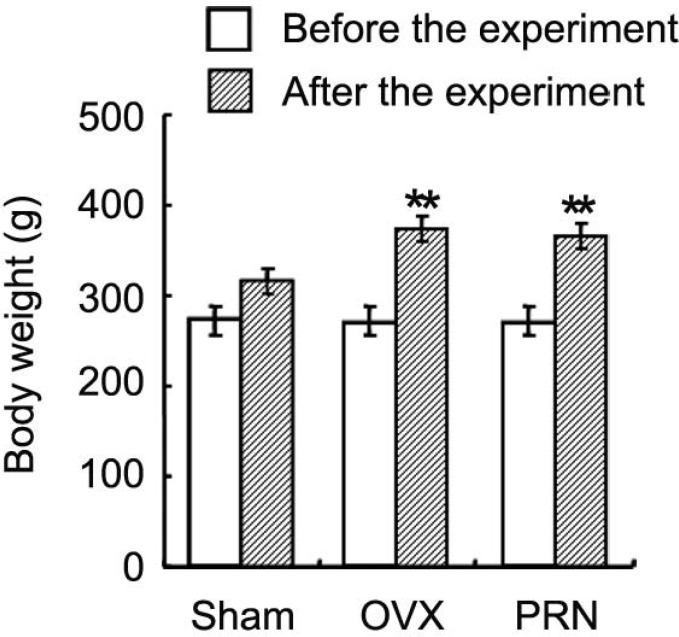
Effect of propranolol on the body weight of animals**. **After 11 weeks administration of propranolol, the body weight of ovariectomized rats was measured. Data are expressed as mean ± SD. (n=10). ** p < 0.01, compared with sham

The body weight of rat in all groups increased from beginning to the end of the experiment (Figure 1). Although the rats in all groups received a similar amount of food, the body weight of rat in OVX (374.1±26.0 g) and PRN (366.6±23.4 g) groups were higher at the end of the experiment than that of sham group rat (315.4±17.2 g). 

*Propranolol increased bone mineral density in ovariectomized rat*

At the end of the experiment, BMD at left femoral and vertebral (the 3^rd^, 4^th^ and 5^th^) was measured by DXA method. In consistent with previous studies, treatment with propranolol increased the BMD at vertebras (Table 1). However, in the present study, BMD increased only at the distal femur in propranolol treatment group (Table 1). No significant difference was observed in total BMD and BMD at the proximal femur between OVX and PRN groups.

**Table 1:** Effect of propranolol on BMD of ovariectomized rats

**Groups **	**BMD (mg/cm** ^2^ **) **					
	**L3 Vertebrae **	**L4 Vertebrae **	**L5 Vertebrae **	**Total Femur **	**Proximal Femur **	**Distal Femur **
Sham	144.3±8.9	150.7±11.0	154.6±7.6	154.4±7.8	155.5±6.5	177.2±13.0
OVX	131.7±7.6	133 4±7.4	137.3±12.1	146.0±4.2^*^	146.1±7.0^*^	158.6±7.9^*^
Propranolol	139.2±6.3^#^	141.9±5.9^# ^	146.5±6.1^##^	150.6±11.7	148.1±8.6	170.6±15.0^##^

At the end of the experiment, the bone mineral density (BMD) was measured by dual energy X-ray absorptiometry. Data are expressed as mean ± SD. (n=10). ^*^ p < 0.05, compared with sham; ^#^ p < 0.05, ^##^ p < 0.01, compared with OVX group.

*Propranolol increased the serum leptin in ovariectomized rat*

**Figure 2:**
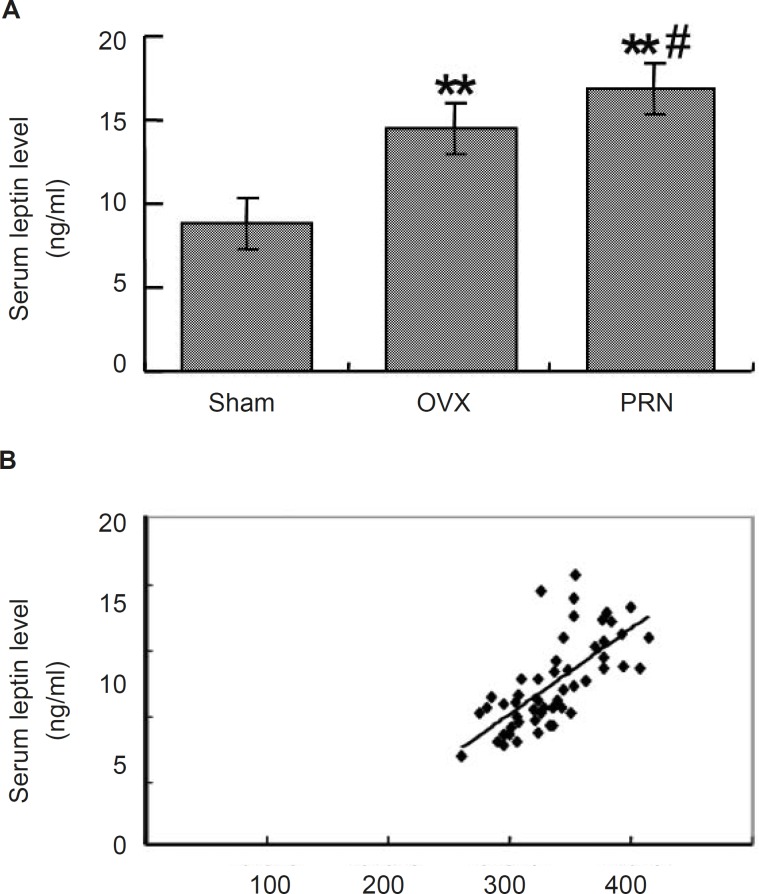
Effect of propranolol on the serum leptin level**. **(A) the serum leptin level of ovariectomized rats (n=10); (B) Linear correlation between the serum leptin and the body weight. Rats were ovariectomized or were treated with sham operation, and then were administrated as mentioned above. Serum leptin levels were examined by ELISA. Data are expressed as mean ± S.D. ^**^ p < 0.01, compared with sham; ^# ^p < 0.05, compared with OVX

To investigate whether the treatment with propranolol could affect the leptin secretion, we measured the serum leptin level by ELISA. As shown in Figure 2A, the leptin level in OVX group (14.502±1.721) increased significantly compared with that of sham group (8.825±0.985). Treatment with propranolol further increased the leptin level (16.875±2.709). When the relationship of the body weight and the leptin level was analyzed, a positive linear correlation was found between the two factors (p < 0.001, r=0.746) (Figure 2B). 

*Propranolol increased leptin receptor level of ovariectomized rat hypothalamus*

**Figure 3:**
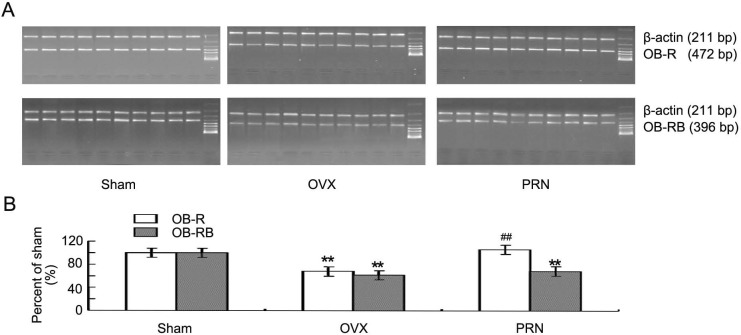
Expression of the hypothalamus leptin receptors mRNA. Both the OB-R and OB-RB mRNA were determined by semi- quantitative RT-PCR. A: The expression of OB-R and OB-RB mRNA was examined by RT-PCR (n=10). Each lane represents the result of one rat. B: Quantities for densitometric analysis of OB-R and OB-RB mRNA. Data are expressed as mean ± S.D. (n=10). ^**^ p < 0.01, compared with sham; ^##^ p < 0.01, compared with OVX

In ovariectomized rats, both the leptin receptor (OB-R) and the long form of leptin receptor (OB-RB) decreased significantly compared with that of sham animals. The OB-R level was up-regulated significantly by propranolol. Although the Ob-Rb expression also increased, there was no significant difference between PRN group and OVX group (Figure 3A and 3B).

## Discussion

The results of the present study confirm the concept that *β*-blockers may be potential drugs for osteoporosis, especially for the postmenopausal women. Propranolol also increased the peripheral leptin level and the leptin receptor level in rat hypothalamus. 

The SNS plays a negative role in bone formation and a positive role in bone resorption. The down regulation of bone formation is dependent on the activation of *β*2-adrenergic receptors, the only *β*-adrenergic receptors known to be expressed by osteoblasts. Therefore, it is hypothesized that the *β*-blockers have a potential role in the treatment of osteoporosis. In previous studies, it has been suggested that *β*-blockers could be used to treat various osteoporoses (12, 13, 15). We also found that propranolol could increase the BMD of ovariectomized rats in this study. This result indicates *β*-blockers may be potential drugs for osteoporosis. 

Leptin, a hormone regulating food intake and energy metabolism exerts its effects mainly through its receptors (OB-Rs) (16), Various variants of the OB-R gene have been identified till now (17, 18). Long form of the OB-R (OB-RB), one of the variants of OB-R gene, is mainly located in the hypothalamus and is considered to be the major form of leptin receptor in the brain (19). OB-Rs are also expressed in other organs and tissues, such as skeletal muscle, liver, and bone (20). It has been shown that leptin could act directly on bone formation and resorption. Subcutaneous injection of leptin reduced the bone loss in ovariectomized rats (10). Leptin treatment upregulated the production of OPG and downregulated Rankl secretion from bone marrow derived stem cells thereby inhibiting the differentiation of osteoclasts (21). In the present study, the peripheral leptin increased in OVX group, and treatment with propranolol could further up-regulate the peripheral leptin level in ovariectomized rats, suggesting a direct role of propranolol on leptin section and subsequent bone metabolism. However, the intracranial injection of leptin could induce a decrease of bone mass in normal mice (22). The deficiency of leptin or its receptors in mice results in a high bone mass phenotype (23, 24). It has been observed that leptin promotes osteoclast differentiation by binding to its receptors in the hypothalamus (7). Leptin stimulates the release of noradrenaline from sympathetic nerve fibers projecting into bone and then inhibits bone formation after noradrenaline binding to *β*2-adrenergic receptors on osteoblasts (7, 23). Therefore, leptin may have two-way adjustment function on the bone mass (25). Our results showed an increase of the leptin receptor level in the hypothalamus by propranolol, indicating an enhanced leptin signaling in the brain. Thus, propranolol controls the leptin signaling in two distinct directions. On one hand, it increased peripheral leptin levels which may promote the bone formation and inhibit the bone resorption; on the other hand, it increased OR-B levels in hypothalamus which may negatively regulate the bone metabolism, whereas the overall outcome of propranolol is upregulation of the BMD of ovariectomized rats. 

Taken together, our results confirmed the anti-osteoporosis role of β-blockers in ovariectomized rats, indicating the *β*-blockers may be used as potential drugs for osteoporosis in postmenopausal women. The effect of propranolol on ovariectomy-induced osteoporosis could be exerted, at least partly, through the regulation of leptin signaling. Additionally, there is an interaction between the SNS and leptin on the regulation of bone metabolism. 

## References

[1] Flier JS (2002). Physiology: is brain sympathetic to bone?. Nature.

[2] Vongpatanasin W, Tuncel M, Mansour Y, Arbique D, Victor RG (2001). Transdermal estrogen replacement therapy decreases sympathetic activity in postmenopausal women. Circulation.

[3] Zoubina EV, Mize AL, Alper RH, Smith PG (2001). Acute and chronic estrogen supplementation decreases uterine sympathetic innervation in ovariectomized adult virgin rats. Histol. Histopathol.

[4] Takeda S, Elefteriou F, Levasseur R, Liu X, Zhao L, Parker KL, Armstrong D, Ducy P, Karsenty G (2002). Leptin regulates bone formation via the sympathetic nervous system. Cell.

[5] Togari A (2002). Adrenergic regulation of bone metabolism: possible involvement of sympathetic innervation of osteoblastic and osteoclastic cells. Microsc. Res. Tech.

[6] Imai S, Matsusue Y (2002). Neuronal regulation of bone metabolism and anabolism: Calcitonin gene-related peptide-, substance P-, and tyrosine hydroxylase-containing nerves and the bone. Microsc. Res. Tech.

[7] Elefteriou F, Ahn JD, Takeda S, Starbuck M, Yang X, Liu X, Kondo H, Richards WG, Bannon TW, Noda M, Clement K, Vaisse C, Karsenty G (2005). Leptin regulation of bone resorption by the sympathetic nervous system and cart. Nature.

[8] Elefteriou F, Takeda S, Ebihara K, Magre J, Patano N, Kim CA, Ogawa Y, Liu X, Ware SM, Craigen WJ, Robert JJ, Vinson C, Nakao K, Capeau J, Karsenty G (2004). Serum leptin level is a regulator of bone mass. Proc. Natl. Acad. Sci.

[9] Evans BA, Agar L, Summers RJ (1999). The role of the sympathetic nervous system in the regulation of leptin synthesis in c57bl/6 mice. FEBS Lett.

[10] Burguera B, Hofbauer LC, Thomas T, Gori F, Evans GL, Khosla S, Riggs BL, Turner RT (2001). Leptin reduces ovariectomy-induced bone loss in rats. Endocrinol.

[11] Sato T, Arai M, Goto S, Togari A (2010). Effects of propranolol on bone metabolism in spontaneously hypertensive rats. J. Pharmacol. Exp. Ther.

[12] Folwarczna J, Pytlik M, Sliwinski L, Cegiela U, Nowinska B, Rajda M (2011). Effects of propranolol on the development of glucocorticoid-induced osteoporosis in male rats. Pharmacol. Rep.

[13] Bonnet N, Benhamou CL, Malaval L, Goncalves C, Vico L, Eder V, Pichon C, Courteix D (2008). Low dose beta-blocker prevents ovariectomy-induced bone loss in rats without affecting heart functions. J. Cell. Physiol.

[14] Pierroz DD, Bouxsein ML, Rizzoli R, Ferrari SL (2006). Combined treatment with a beta-blocker and intermittent pth improves bone mass and microarchitecture in ovariectomized mice. Bone.

[15] Zhang W, Kanehara M, Zhang Y, Wang X, Ishida T (2007). Beta-blocker and other analogous treatments that affect bone mass and sympathetic nerve activity in ovariectomized rats. Am. J. Chin. Med.

[16] White DW, Kuropatwinski KK, Devos R, Baumann H, Tartaglia LA (1997). Leptin receptor (ob-r) signaling. Cytoplasmic domain mutational analysis and evidence for receptor homo-oligomerization. J. Biol. Chem.

[17] Considine RV, Considine EL, Williams CJ, Hyde TM, Caro JF (1996). The hypothalamic leptin receptor in humans: identification of incidental sequence polymorphisms and absence of the db/db mouse and fa/fa rat mutations. Diabetes.

[18] Chung WK, Power-Kehoe L, Chua M, Chu F, Aronne L, Huma Z, Sothern M, Udall JN, Kahle B, Leibel RL (1997). Exonic and intronic sequence variation in the human leptin receptor gene (lepr). Diabetes.

[19] Baskin DG, Schwartz MW, Seeley RJ, Woods SC, Porte D, Breininger JF, Jonak Z, Schaefer J, Krouse M, Burghardt C, Campfield LA, Burn P, Kochan JP (1999). Leptin receptor long-form splice-variant protein expression in neuron cell bodies of the brain and co-localization with neuropeptide y mrna in the arcuate nucleus. J. Histochem. Cytochem.

[20] Hamrick MW, Ferrari SL (2008). Leptin and the sympathetic connection of fat to bone. Osteoporos Int.

[21] Holloway WR, Collier FM, Aitken CJ, Myers DE, Hodge JM, Malakellis M, Gough TJ, Collier GR, Nicholson GC (2002). Leptin inhibits osteoclast generation. J. Bone Miner. Res.

[22] Takeda S, Karsenty G (2001). Central control of bone formation. J. Bone Miner. Metab.

[23] Ducy P, Amling M, Takeda S, Priemel M, Schilling AF, Beil FT, Shen J, Vinson C, Rueger JM, Karsenty G (2000). Leptin inhibits bone formation through a hypothalamic relay: A central control of bone mass. Cell.

[24] Karsenty G (2001). Leptin controls bone formation through a hypothalamic relay. Recent Prog. Horm. Res.

[25] Karsenty G (2006). Convergence between bone and energy homeostases: Leptin regulation of bone mass. Cell Metab.

